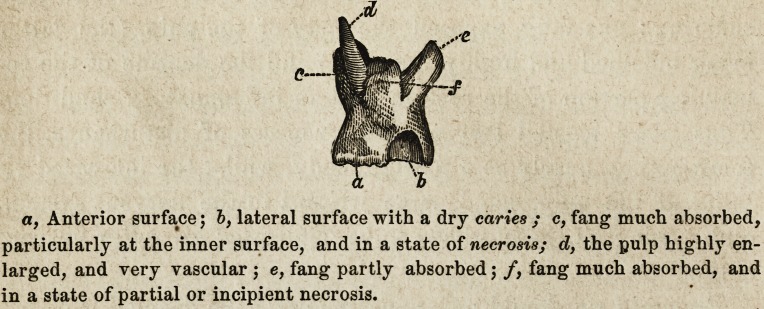# Interesting Case of Hemicrania, from a Carious Tooth and Necrosed Fangs, with a Highly Vascular Condition of the Pulp-Cavity

**Published:** 1857-10

**Authors:** J. L. Levison


AMERICAN JOURNAL

OF

DENTAL SCIENCE.

Vol. VII. NEW SERIES—OCTOBER, 1857. No. 4.

### ORIGINAL COMMUNICATIONS.

### ARTICLE I.

*Interesting Case of Hemicrania, from a Carious Tooth and Necrosed Fangs, with a highly Vascular Condition of the Pulp-cavity.* (With a drawing.) By J. L. Levison, D. D. S., &c.

Mr. N——, a gentleman of a nervo-bilious temperament, of sedentary habits, rather above the age of fifty, had frequent neuralgic attacks affecting the half of the face, head and side; and under some circumstances to be incidentally mentioned, the paroxysms of pain in the jaw, from the glenoid cavity to the antrum, was at times maddening. These symptoms were sure to be aggravated, whenever their existed hard feculent matter in the alimentary canal, or any source of irritation to the mucous tissues of the body.*

Hence, when the alvine secretions were normal and regular, he had an impunity from suffering: but when from excitement

* This source of odontalgia might have been anticipated, *a priori*, when we reflect that the rudimentary teeth are in their first stage—mucous follicles.

or too great mental labor, these functions were so much deranged that constipation of the bowels was the result—then his whole moral being was disturbed. Under these disturbing influences, he was attacked with a series of painful shocks, like a discharge from the Leyden jar—the pain being excruciating, first in the tooth, from which it seemed to dart to the alveolus; and then, like a demoniac-spirit, it would rush to the angle of the jaw, from thence to the temples, where the cerebral hemispheres responded with a dull, heavy pain, and a sense of fulness of the blood vessels within and without the cranium, which denotes a state of congestion. And besides these well marked *data*, there existed another equally important as a diagnostic indication of functional disturbances, namely, that there was a higher temperature on the affected side of the head, the greater sensible heat, being most palpable to the touch. The face being at the same time actually flushed, and often the one eye blood-shot.

The principal remedy the patient tried for some relief, was to smoke tobacco, which by narcotising the affected nerves often allayed the pain, but this was nevertheless partial and uncertain in its consequences. On some states of his system smoking only aggravated the symptoms, and rendered the attack almost unendurable.

We may remark, *en passant*, that this gentleman had a very large *organ of firmness*, and thus he persevered against his better sense, by putting off the evil day, but in a moment of frenzy he had it extracted. And we have deemed it worthy of an especial notice as a most well marked case of true hemicrania.

When the tooth is examined, even through the means of the accompanying drawing, made within an hour of its extraction, the marvel will not be that our patient suffered, but that there could be experienced actual freedom from pain for a month or two at a time, and then a renewal of the horrid torture.

One reason may be given to account for this apparent anomaly: Mr. N—— was a temperate man in eating and drinking, and generally attended to the laws of health.

The tooth was not decayed on the anterior surface of the crown, but on one of the lateral sides, but that only superficial; the *caries* not having penetrated the pulp cavity. But the fangs were the seat of the mischief and the source of the intense suffering: one fang exposed the body of the pulp, (the latter being inflamed and highly vascular,) and the dentine of the remaining portion of the root showed an inflammatory condition. Thus there existed two powerful sources of disturbance, the tooth acting nearly as a foreign body, whilst the increased vitality of the pulp, acted as an irritant to the nervous system, and causing all these organs to be implicated by reflex nervous action.

Probably we should not, nevertheless, have deemed it of sufficent importance to give it a place in your excellent Journal, but from the fact that it confirms a theory we propounded on the disease of the fangs, incidental, to all who smoke tobacco in extreme, and which our patient certainly did. [See Medical Times, December 4th, 1849, an article entitled "on certain effects of an inveterate habit of smoking—the denudation of the periosteum of the fangs of the teeth, by J. L. Levison."] In the case we have narrated in this short paper, the patient had been a great smoker, but for some few years prior to the diseased symptoms we have mentioned, he had not indulged to excess—he was a regular consumer of the Indian weed, and in the tooth (see drawing) there was a similar peculiarity as mentioned in the paper in the Medical Times, a denudation of the periosteum of the fangs. Finally, we consider, from many years observation, that there is never any active absorption of the fangs, until the periosteum is denuded.*

19 Dorset Place, Dorset Square, London, *June 10th*, 1857.

* We showed this paper to our patient, and he complimented us for the rigid accuracy of our report, but adds that he continues to smoke in moderation and to confine himself to water drinking, and he feels assured he will never lose another tooth.

*An Upper Molar Tooth, extracted from a gentleman of sedentary habits, whose health suffered from it.*

*a,* Anterior surface; *b,* lateral surface with a dry *caries;* *c,* fang much absorbed, particularly at the inner surface, and in a state of *necrosis;* *d,* the pulp highly enlarged, and very vascular; *e,* fang partly absorbed; *f,* fang much absorbed, and in a state of partial or incipient necrosis.

---

## Figures and Tables

**Figure f1:**